# Adolescent Neural Reactivity to Alcohol Cues: The Role of Violence Exposure and Coping Motives

**DOI:** 10.3390/bs16020218

**Published:** 2026-02-03

**Authors:** Kathryn C. Jenkins, Alexa House, Kayla Kreutzer, K. Luan Phan, Stephanie M. Gorka

**Affiliations:** 1 Institute for Behavioral Medicine Research, The Ohio State University, 460 Medical Center Drive, Columbus, OH 43210, USA; lexihouse8@gmail.com (A.H.); kayla.kreutzer@osumc.edu (K.K.); stephanie.gorka@osumc.edu (S.M.G.); 2 Neuroscience Graduate Program, The Ohio State University, Columbus, OH 43210, USA; 3 Department of Psychiatry and Behavioral Health, The Ohio State University Wexner Medical Center, 370 W. 9th Avenue, Columbus, OH 43210, USA; luan.phan@osumc.edu

**Keywords:** exposure to interpersonal violence, alcohol cue reactivity, late-positive potential (LPP), drinking to cope

## Abstract

Exposure to violence (physical, domestic, or sexual assault) increases risk for alcohol problems and alcohol use disorder (AUD), consistent with self-medication and drinking-to-cope theories of alcohol use, which posit that some individuals may misuse alcohol to alleviate distress associated with trauma. Yet how violence exposure and coping motives interact to influence objective AUD risk markers remains unclear. Emerging evidence suggests that trauma type affects psychiatric outcomes, but its role in moderating AUD risk via coping motives remains unknown. We examined these gaps in the literature in a cohort of youth (ages 16–19; *n* = 157) over-sampled for violence exposure. Participants completed a structured trauma interview and an assessment of drinking motives. A total of 60 participants reported experiencing sexual assault (SA), 54 physical assault (PA), and 32 domestic violence (DV). AUD risk was captured using the alcohol cue reactivity paradigm. Participants were exposed to images of alcoholic beverages, high-calorie foods (reward-related control), and neutral objects. The late positive potential (LPP), an event-related potential captured via electroencephalogram, was used to index cue reactivity. We ran two linear regression analyses to assess the relationship between trauma type and coping motives to drink on LPP to alcohol and food cues (>neutral). For alcohol cues, there was a significant SA and coping interaction. At high levels of coping motivations, SA was associated with enhanced LPP to alcohol cues. At low levels of coping motivations there was no association. No effects were observed for food cues. Our results demonstrate that heightened coping motives to drink are associated with enhanced alcohol cue reactivity among SA victims, indicating increased vulnerability for AUD risk.

## 1. Introduction

Exposure to interpersonal violence is common, especially among adolescents, with nearly two-thirds of U.S. youth experiencing at least one form of direct or indirect violent experience each year (Finkelhor et al., 2015). Among youth, 60.8% report being victims of violent acts, including physical assault (PA), sexual assault (SA), and witnessing home domestic violence (DV) (Finkelhor et al., 2015). These statistics are particularly concerning given the strong link between interpersonal violence exposure and heightened vulnerability to a variety of mental health disorders, including depression, anxiety, and post-traumatic stress disorder (PTSD), as well as substance and alcohol use disorders (SUD/AUD) (Covey et al., 2020; Carliner et al., 2016; McLaughlin & Lambert, 2017). Importantly, research indicates that individuals who experience interpersonal violence are two to three times more likely to develop alcohol-related problems compared to those without a history of trauma (Harrison et al., 1997; Berenz et al., 2016). This association is particularly concerning given that adolescence is a developmental period associated with increased risk-taking behaviors and alcohol misuse (E. J. Marshall, 2014). It is therefore critical that we investigate the underlying mechanisms that drive the association between exposure to violence and alcohol problems to inform targeted prevention and intervention efforts in high-risk youth.

Numerous theories have been proposed to explain the association between exposure to violence and risk for AUD. A prominent theory is the “drinking-to-cope” model (Cooper et al., 1995, 2016), which posits that some individuals may use alcohol alleviate heightened distress following exposure to traumatic stressors such as violence victimization (Hawn et al., 2020a). Violence victimization often heightens psychological distress—manifesting as anxiety, depression, and post-traumatic stress—and individuals who rely on alcohol to cope may begin to self-medicate with alcohol (Khantzian, 1997; Conger, 1956). While the anxiolytic effects of alcohol consumption may offer temporary relief, habitual drinking to cope reinforces maladaptive behaviors, ultimately increasing the risk for AUD (Koob & Le Moal, 2001; Hawn et al., 2020a). Thus, exposure to violence is a robust, acute stressor that potentiates risk for AUD. However, not all individuals who experience interpersonal violence endorse coping motives to drink or engage in alcohol use (Bountress et al., 2019). This suggests that only certain subsets of individuals are vulnerable to AUD in the aftermath of violence exposure and that the ‘drinking-to-cope’ model may characterize only specific trajectories of AUD risk. Identifying the individuals most at risk for this maladaptive pathway is essential for developing targeted interventions aimed at preventing the onset and escalation of alcohol misuse. By pinpointing key demographic, psychological, and neurobiological factors that contribute to heightened vulnerability, we can tailor prevention and treatment efforts to those who need them most, ultimately improving outcomes for at-risk populations.

Trauma type may play a critical role in shaping an individual’s risk for AUD. Growing evidence suggests that the nature of a traumatic experience significantly influences the development of psychiatric symptoms following victimization (McLaughlin & Lambert, 2017; H. L. Smith et al., 2016). Indeed, research has shown that distinct trauma profiles are associated with varying symptom presentations (Bryant et al., 2013; Levin et al., 2021), highlighting the nuanced impact of trauma on mental health outcomes. Broadly, interpersonal violence exposure, defined as SA, PA, or DV, is associated with particularly high rates of AUD, relative to non-interpersonal violence such as accidents and natural disasters (Stangeland et al., 2023; Levin et al., 2021). However, much of the existing literature has relied on broad, dichotomous groupings of mixed trauma types, which, while affording increased statistical power, can obscure important effects of distinct trauma experiences. Increasingly, studies have begun to focus on how different facets of trauma may uniquely contribute to risk for AUD and problem drinking (Bitsoih et al., 2023; Noudali et al., 2022; Patock-Peckham et al., 2020). Among interpersonal traumatic experiences, SA in particular appears to show a strong and consistent association with alcohol misuse and AUD (Levin et al., 2021; Rich et al., 2016). This is especially concerning given that SA is a pervasive problem among adolescents and young adults in the United States (Berzofsky et al., 2013; Muehlenhard et al., 2017; Basile et al., 2022). Notably, this developmental period also coincides with the initiation of alcohol use (Chen & Yoon, 2021) as well as increased incidence of risky behaviors such as binge drinking, which further increases the risk of AUD (Chung et al., 2018). Taken together, adolescence represents a period of vulnerability in which both exposure to SA and the onset of problem drinking tend to occur. In fact, the literature has provided evidence that SA may serve as a reliable indicator of heightened risk for AUD (Ullman, 2003; Sartor et al., 2008; Langdon et al., 2017), suggesting that youth who have experienced SA victimization may be uniquely vulnerable to alcohol-related problems. Nevertheless, only a handful of studies have examined if and how different types of trauma may interact with other known risk factors for problem drinking among youth, leaving critical gaps in our understanding of who is most at risk.

Motivation to consume alcohol is another established risk factor for AUD that plays a critical role in the ‘drinking-to-cope’ framework. Theory and research posit that alcohol use is guided by one’s motivation to drink, influenced by internal and external incentives to modulate affective states through alcohol consumption (Cox & Klinger, 1988). Drinking motivations exist as both stable traits reflecting an individual’s propensity for drinking as well as dynamic states that fluctuate given the context of a situation. Other factors, such as personality traits, have also been linked to drinking frequency in adolescents, highlighting how stable traits can shape drinking behavior (Kang, 2023). Based on Kox and Clinger’s model, there are at least four primary motivations to drink: enhancement motives, coping motives, social motives, and conformity motives. There is substantial evidence that adolescents who endorse coping-motivated alcohol use are more likely to develop problematic drinking patterns in the future, relative to adolescents who endorse other motivations for drinking (Stapinski et al., 2016; Cooper et al., 2016; Zaso et al., 2023). In addition, coping motives for drinking have been found to moderate the relationship between heightened distress and heavy alcohol use (K. Z. Smith et al., 2014; Simpson et al., 2014). Importantly, this finding has been recapitulated in the context of SA; one study found that drinking to cope moderates the relationship between SA-related distress and problem drinking (Stappenbeck et al., 2015). However, this study was conducted in a sample of biological women with a history of SA only and did not examine if and how this relationship can differ across different trauma types. Supporting the relevance of examining different trauma types, Patock-Peckham et al. (2020) found that SA was associated with dysregulated drinking in men as opposed to females, highlighting that certain trauma experiences within specific groups may uniquely influence alcohol-related risk. It may be that among victims of SA, coping-related motivation for alcohol use is a particularly salient risk factor for AUD and alcohol problems as opposed to other trauma types, though this has not been directly tested.

Brain-based laboratory paradigms can provide objective indices of AUD risk. One existing, well-established marker of alcohol problems is enhanced alcohol cue reactivity. This is identified using the alcohol cue reactivity task (Carter & Tiffany, 1999), which exposes participants to alcohol-related stimuli and captures subjective and objective alcohol craving responses. Studies have shown that heightened self-reported craving correlates with AUD severity, predicts subsequent alcohol relapse, and worsens treatment outcomes (Sinha, 2011; Schneekloth et al., 2012; see Vafaie & Kober, 2022 for review). Beyond self-report, one objective neural marker of alcohol cue reactivity is the late positive potential (LPP). The LPP is an event-related potential measured using an electroencephalogram (EEG) and is observed around 400 milliseconds after presentation of a visual stimulus over occipital-parietal sites. The LPP is a psychometrically robust marker of motivated attention and salience processing across emotional and substance-related picture paradigms (Brown et al., 2012; Hajcak et al., 2009). Drug vs. neutral cues reliably elicit greater LPP in those with SUDs relative to controls (see Littel et al., 2012 for review). Enhanced alcohol cue reactivity has also been observed in those with higher relative to lower AUD risk profiles (Bartholow et al., 2007), as well as in binge drinkers (Almeida-Antunes et al., 2021). Further, greater neural alcohol cue reactivity has been shown to predict the speed and intensity of alcohol consumption during real-life episodes of drinking (Kohen et al., 2024). While neural markers such as the LPP are less clinically feasible than self-report measures, they can provide insight into early occurring motivational processes that may not always be captured through subjective reporting. Recent work from our lab further supports this predicative utility. Jenkins et al. (2025) showed that, in this same youth sample, individual differences in LPP to alcohol cues are present very early on in youth’s drinking trajectories and can be used to reliably predict increases in future drinking behaviors while controlling for baseline drinking exposure. These results demonstrate that in youth, the LPP is not merely a correlate of existing risky alcohol use but rather an early occurring risk marker that precedes escalations in drinking behavior. Collectively, an enhanced LPP to alcohol cues is a well-validated objective indicator of risk for the development of alcohol problems and AUD.

Exposure to interpersonal violence is a well-established risk factor for AUD, and yet it remains unclear who is most at risk for developing alcohol problems and why and how those individuals are vulnerable. The present study examined this question in a cohort of adolescents with a range of complex interpersonal trauma histories who had minimal alcohol exposure but were at high-risk for the development of alcohol-problems. This sample is uniquely suited to address this gap in the literature, capturing the developmental period during which drinking behaviors are established in an at-risk population oversampled for trauma exposure. We hypothesized that among victims of SA, those with heightened drinking-to-cope motivations would display an enhanced LPP to alcohol cues.

## 2. Methods

### 2.1. Participants

Participants were recruited as part of a larger study that aimed to examine the neurobiological mechanisms underpinning the association between interpersonal traumatic experiences and risk for AUD. Participants were recruited from the Columbus, Ohio area through flyers posted locally, social media advertisements, and through recruitment efforts at local high schools and colleges. Participants were required to be between the ages of 16 and 19 and had to provide written informed consent or assent along with parental consent. Participants were recruited based on trauma history and assigned to one of two groups: (1) individuals with a lifetime history of interpersonal trauma exposure and (2) individuals with no such history. Given that the larger study for which individuals were recruited aimed to examine initial onset of AUD, participants were required to have had minimal alcohol consumption upon enrollment (i.e., self-reported consumption of >1 but <100 standard alcoholic drinks in their lifetime) but be at risk for the development of alcohol problems (e.g., surrounded by risky peers and reporting access to alcohol). Exclusionary criteria included any major active medical or neurological illness, lifetime history of hypomania/mania/psychosis, deafness, traumatic brain injury, current psychiatric medication use, lifetime history of alcohol or substance use disorder, and pregnancy. At baseline, participants completed a virtual screening session that included informed consent and assent, clinical diagnostic interviews, and self-report questionnaires. Additionally, participants completed a baseline EEG for which they were instructed to abstain from drugs/alcohol at least 48 hrs prior to the lab assessment. This was verified via breath alcohol and urine drug screens. A total of 157 participants (111 females; 46 males) were included in the present study. All participants were monetarily compensated for their time. All study procedures were approved by The Ohio State University Institutional Review Board.

### 2.2. Clinician- and Self-Administered Assessments

Upon enrollment, participants completed an initial screening session that included a battery of well-validated self-report questionnaires and a structured clinical interview administered by trained assessors. Participants who were 18 years old or older completed the Structured Clinical Interview for the DSM-5 (SCID-5; First et al., 2015). Participants under the age of 18 completed a modified version of the Kiddie Schedule for Affective Disorders and Schizophrenia (K-SADS; Chambers et al., 1985).

### 2.3. UCLA Post-Traumatic Stress Disorder (PTSD) Reaction Index (RI)

Lifetime history of interpersonal trauma exposure was captured using the trauma screener from the UCLA PTSD Reaction Index (RI) for DSM-5 (Steinberg et al., 2004). The UCLA PTSD RI is administered by a clinician and designed to capture a wide range of traumatic and stressful life experiences in both children and adolescents. The screener encompasses 18 different traumatic and stressful life events: neglect/maltreatment, sexual abuse, sexual assault/rape, physical abuse, interpersonal violence, emotional abuse, domestic violence, community violence, war/political violence, life-threatening medical illness, serious accident, school violence, disaster, terrorism, kidnapping, bereavement, separation from caregiver, and impaired caregiver. For the present analyses, sexual abuse and sexual assault/rape were collapsed into one SA variable to ensure adequate sample size for analyses, as the individual would be too small to be examined separately. Likewise, the PA variable was created by collapsing physical abuse, defined as abuse perpetrated by a family member, and interpersonal violence, defined as assaultive events occurring outside of the family (i.e., a stranger, a friend, a peer). These categories reflect conceptual overlap, and prior studies using the UCLA have similarly combined related trauma items when conceptually aligned (e.g., Cole et al., 2016; Pynoos et al., 2014). All responses were dichotomous and made on a yes-versus-no checklist. As such, variables were coded as follows: 0 = no trauma exposure, 1 = trauma exposure.

### 2.4. Post-Traumatic Stress Disorder (PTSD) Checklist for DSM-5 (PCL-5)

The PCL-5 is a 20-item self-report measure that evaluates the degree to which an individual has experienced PTSD symptoms in the past month related to a distressing experience (Weathers et al., 2013). The PCL-5 consists of four subscales, each of which captures the four DSM–5 PTSD symptom clusters: intrusion (Items 1–5), avoidance (Items 6–7), alterations in cognition and mood (Items 8–14), and hyper arousal and reactivity (Items 15–20). Items are rated from 0 (not at all) to 4 (extremely) and were summed to create a total severity score. Internal consistency for the PCL was excellent, α = 0.92.

### 2.5. Drinking Motives Questionnaire Revised (DMQ-R)

Drinking motivations were assessed using the Drinking Motives Questionnaire Revised (DMQ-R; Grant et al., 2007; adapted from Cooper, 1994), a 28-item self-report measure designed to evaluate different motivations for alcohol consumption. The DMQ-R assesses five primary drinking motivations: social, enhancement, conformity, coping–anxiety, and coping–depression. Participants rated how frequently they drink for each respective reason on a 5-point Likert scale ranging from 1 (almost never/never) to 5 (almost always/always). Higher scores indicated a stronger endorsement. Consistent with prior research and theoretical models, the present study combined and averaged the coping–depression and coping–anxiety subscales to form a single drinking-to-cope composite score (Bravo & Pearson, 2017; Corbin et al., 2013; Shuai et al., 2022). These subscales have been found to be highly correlated and are thought to reflect overlapping negative reinforcement processes. Additionally, the present study did not have specific hypotheses regarding differential effects of coping–anxiety versus coping–depression but rather, the focus was on broader negative reinforcement processes. Internal consistency was α = 0.80 for the coping–anxiety subscale, α = 0.93 for the coping–depression subscale, and α = 0.93 for the combined scale.

### 2.6. Cue Paradigm

Participants participated in a picture viewing task modified from prior studies on cue reactivity (Demos et al., 2011; Wagner et al., 2012). The task consisted of three image types: alcoholic beverages, high-calorie foods (a reward-related control), and neutral objects. Alcohol and food images were adapted from prior published studies (e.g., Wagner et al., 2012) and were presented on a white background. Neutral images were selected from the International Affective Picture System (IAPS; Lang, 2005). Participants viewed 2 blocks of 30 alcohol images, 30 high-calorie food images, and 30 neutral objects for a total of 180 images. Each block presented photos to participants in a randomized fashion. Images were presented to participants for 2500 ms, followed by an interstimulus interval of 2000 ms. Intermittently, participants were prompted to quickly respond with the mouse to a question mark to indicate that they were maintaining attention during the task. Question marks appeared on average around 30 times throughout the entire task. The total task duration was 12 min.

### 2.7. Electroencephalogram Data Collection and Processing

**Figure 1:**
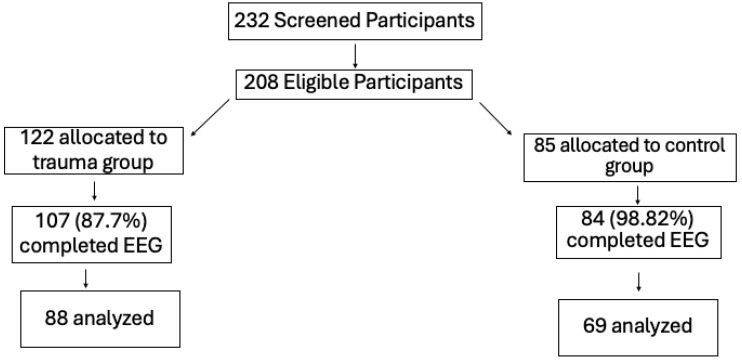
Diagram illustrating participant flow through the study, including enrollment and inclusion in the final analyses.

Continuous EEG was recorded using the ActiveTwo BioSemi system (BioSemi, Amsterdam, The Netherlands). We utilized thirty-four standard electrode sites, and one electrode was placed on each mastoid. Brain Vision Analyzer 2 software was used to perform off-line analyses (Brain Products, Gilching, Germany). Data was digitized using a sampling rate of 1024 Hz and a low-pass fifth-order sinc filter with a −3 dB cutoff point at 208 Hz. Data processing was performed through EEGLAB (Delorme & Makeig, 2004) and ERPLAB (Lopez-Calderon & Luck, 2014) within the MATLAB environment (Mathworks, 2022a). Raw EEG signals were resampled to 256 Hz using an antialiasing filter, re-referenced to the average mastoid electrodes, and bandpass filtered using a Butterworth filter (range of 0.10–30 Hz). EEGLAB was programmed to identify and remove channels exhibiting excessive noise, which was defined as more than 4 standard deviations above the mean channel noise, prolonged flatlining (over 5 s), and or poor correlation with neighboring channels (<0.85) (clean_rawdata, Kothe & Makeig, 2013). All channels removed were subsequently interpolated, and data were segmented beginning 200 ms before stimulus presentation and continuing for 2500 ms for the full presentation of the stimulus. Baseline correction for each trial was performed using the 200 ms prior to stimulus onset and was averaged across each condition. Participants were required to have a minimum of 12 artifact-free trials in each condition to be included in analyses (Moran et al., 2013). After pre-processing, an average of 74.73% alcohol, 72.51% food, 71.67% neutral trials were accepted. 25 participants did not meet the requirements for artifact free trails and were removed from the analysis. Therefore, 16% of the sample was lost due to artifact-contaminated trials. A diagram has been included to illustrate participant flow from enrollment through inclusion in the final sample (see Figure 1). The LPP was scored as the mean activity from 400 to 2500 ms at parietal site Pz for each image type. This approach was not pre-registered but was driven by previous studies (Herrmann et al., 2000; Bartholow et al., 2007; Jenkins et al., 2025) and based on the visual inspection of our data as to where the LPP was maximal. A higher LPP can be interpreted as a greater neural response to cued images, whereas a lower LPP is interpreted as a smaller neural response to cued images.

### 2.8. Data Analysis Plan

To test our hypotheses, we conducted a series of hierarchical linear regression analyses. LPP to alcohol cues (>neutral cues) was entered as the dependent variable. Biological sex, age, and PCL-5 total symptoms were included as covariates in block 1. These covariates were selected because age and biological sex have been shown to influence alcohol cue reactivity (Radoman et al., 2024; Kaag et al., 2019). PCL-5 symptoms were included to capture PTSD symptom severity and isolate the relative impact of trauma type. Coping motives to drink, SA, PA, and DV victimization were entered into block 2. Two-way interaction terms between trauma type (SA, PA, and DV) and coping motives to drink were created and entered in block 3. Significant two-way interactions were followed up using a standard simple slopes approach (Aiken et al., 1991). We created two new conditional moderators at high and low levels of the drinking motives by adding and subtracting 1 SD from the mean. Follow-up analyses were run at high and low levels of drinking-to-cope motives.

We also conducted a sensitivity analysis to assess whether our findings extended to neural responses to other reward-related stimuli, specifically high-calorie foods. We re-ran the model described above using LPP to food cues (>neutral cues) as the dependent variable. All analyses were conducted using SPSS v27 (IBM).

## 3. Results

### 3.1. Clinical and Demographic Characteristics

Participant descriptives and characteristics are presented in Table 1. On average, participants endorsed exposure to 3.14 ± 2.4 traumatic events in their lifetime. PCL total scores ranged from 0 to 59. The most commonly endorsed traumas were bereavement (42%), sexual violence (abuse or assault; 37.6%), physical abuse (abuse or assault; 34.4%), emotional abuse (33.1%), and domestic violence (19.7%). A total of 38 individuals met criteria for lifetime PTSD (24.1%), and 7 individuals met criteria for current PTSD (4.4%). At baseline, participants reported an average of 36.29 (+/−28.17) alcoholic beverages consumed in their lifetime. No participants met the criteria for alcohol or substance use disorders. Among participants who reported SA (*n* = 60), 28 (46.67%) endorsed PA and 15 (25%) reported DV. Lastly, the two subscales comprising the drinking-to-cope composite score, drinking-to-cope with anxiety and drinking to cope with depression, were strongly correlated (r = 0.77, *p* < 0.001). Scores on the composite drinking to cope scale ranged from −0.83 to 5.52. Model diagnostics indicated that assumptions for linear regression were adequately met. Outlier analysis using Cook’s Distance revealed no cases exceeding 1.0, suggesting no single data point unduly influenced results. A histogram of standardized residuals approximated a normal distribution, with minimal skew or kurtosis, and residuals plotted against predicted values were randomly dispersed around zero, supporting the assumptions of normality, linearity, and homoscedasticity.

### 3.2. Trauma Types and Coping Motivations on LPP to Alcohol Cues

**Figure 2:**
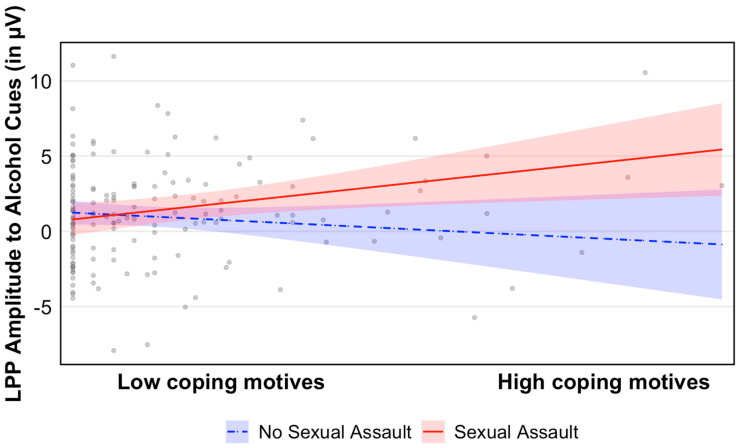
Graph displaying the effects of low and high drinking-to-cope motives on LPP to alcohol cues among victims and non-victims of SA. No exposure to sexual assault (*n* = 98); exposure to sexual assault (*n* = 60). Values reflect model-adjusted predicted estimates. Although coping motives were specified as the moderator in the statistical model, the sexual assault group is displayed for visualization purposes.

Results for the omnibus model are presented in Table 2. Our results indicated that there was a main effect of PTSD symptom severity on LPP to alcohol cues, such that greater PTSD symptoms were associated with an enhanced LPP amplitude to alcohol cues. We found no main effects of age, biological sex, SA, PA, DV, or coping motives to drink on LPP to alcohol cues. Our findings revealed a significant two-way interaction between SA and coping motives to drink (b = 1.113, t = 2.425, *p* = 0.017). Follow-up analyses revealed that at high levels of drinking-to-cope motivations, SA was associated with an enhanced LPP to alcohol cues (b = 1.633, SE = 0.803, *p* = 0.044). However, at low levels of drinking-to-cope motivations, there was no association between SA and LPP to alcohol cues (b = −1.136, SE = 0.825, *p* = 0.171) (see Figure 2). We found no significant interactive effects of PA, DV, and coping motives on LPP to alcohol cues.

### 3.3. Trauma Types and Coping Motivations on LPP to Food Cues

As a sensitivity analysis, we examined whether our results extended to neural responses to non-alcohol reward cues, using LPP amplitude to high-calorie foods. Our results indicated no main or interactive effects of trauma symptoms, trauma type, and coping motives to drink on LPP to food cues.

## 4. Discussion

The aim of the present study was to investigate the role of trauma type and drinking motivations in risk for AUD and problematic alcohol use. This study was informed by self-medication and drinking-to-cope theories of alcohol use, which propose that coping-motivated drinking may contribute to trauma-related distress and risk for alcohol problems (Khantzian, 1997; Baker et al., 2004). Within this theoretical framework, our results revealed an interaction between SA and drinking-to-cope motivations, even after controlling for PTSD symptoms. This association was specific to SA victimization and did not emerge in other trauma types or to non-alcohol reward-related control cues. Taken together, our results suggest that heightened drinking-to-cope motivations are associated with enhanced neural reactivity to alcohol cues among SA survivors, indicating potential increased vulnerability for AUD and alcohol problems in this high-risk subgroup.

Our results revealed that at high levels of drinking-to-cope motives, SA victimization is associated with enhanced LPP to alcohol cues. This pattern suggests that among individuals with heightened drinking-to-cope motivations, a history of SA is significantly associated with increased risk for developing AUD. In contrast, SA victims who reported low levels of drinking-to-cope motivations did not display enhanced alcohol cue reactivity, implying that SA alone is not a sufficient predictor of AUD development. Rather, vulnerability appears to emerge when sexual trauma exposure is paired with the use of alcohol as a coping strategy. These findings highlight coping motives as a key vulnerability factor that can determine whether trauma exposure translates into the development of alcohol-related problems. This interpretation is consistent with self-medication or negative reinforcement models of AUD risk (Hawn et al., 2020a; see Hawn et al., 2020b for review, Khantzian, 1997; Conger, 1956). These results are also in line with the recent literature, which displays that the different facets of trauma, particularly sexual abuse, can contribute to alcohol misuse through coping-motivated drinking strategies (Bitsoih et al., 2023). However, it is also important to consider the possibility that some individuals may have exhibited elevated coping motives prior to trauma exposure, which could in turn increase their likelihood of encountering high-risk situations. Consistent with this perspective, research has found that drinking-to-cope motivations are extremely common among adolescents and young adults and are reliably associated with higher levels of alcohol consumption, binge drinking episodes, and negative alcohol-related consequences (Park & Levenson, 2002; Smit et al., 2022; Stapinski et al., 2016). In addition, one study found that greater coping motivations for drinking and emotional dysregulation were robust predictors and consequences of instances of SA among college-aged females (Messman-Moore et al., 2014). This is highly important as it highlights a critical gap in our understanding of who is most at risk and how these risk factors unfold over time. Future research should seek to clarify the temporal nature of this relationship in order to better understand the context in which these factors interact to elevate risk for alcohol problems.

Interestingly, PA and DV victimization were found to be unrelated to alcohol cue reactivity. This suggests that there may be certain characteristics inherent to SA that influence AUD risk and neural response to alcohol cues. One possibility is that SA may uniquely alter how individuals engage with distress and utilize reinforcement-based coping strategies, thus strengthening the association between negative affect and alcohol use. Sexual assault is often regarded as one of the most severe interpersonal traumas and can fundamentally disrupt beliefs about one’s individual safety and trust (see Dworkin et al., 2017 for review; Dworkin, 2020). Such disruptions may keep individuals in a chronic state of anticipatory anxiety and elevated hypervigilance resulting from fear of re-occurrence of danger and victimization. When elevated stress is coupled with heightened drinking-to-cope motives, the risk for problematic alcohol use may be amplified (Windle & Windle, 2015; Geda et al., 2024). Although PA and DV can be comparable to SA in terms of severity and impact on emotional well-being, they may not produce the same alterations that reinforce the salience of alcohol cues in the presence of coping motivations. Finally, it is important to acknowledge that we cannot fully disentangle biological sex from trauma type in the present study, as victims of SA are disproportionately biological females (Cal Poly Humboldt, 2025). Additionally, a substantial body of work has demonstrated that females exhibit a heightened vulnerability to developing PTSD following a traumatic event as opposed to males (see Haering et al., 2024, for review). At the same time, SA in males has been found to be indirectly linked to alcohol problems through elevated PTSD symptoms and impaired control (Patock-Peckham et al., 2020), and drinking-to-cope motivations are strongly associated with problem drinking among males with heightened depressive symptoms (Foster et al., 2014). This raises the possibility that biological sex could be an important factor that influences coping motivations and alcohol-related outcomes. Future studies should attempt to directly probe the role of biological sex in this risk pathway to better clarify who is most at risk for the development of alcohol problems.

These findings have important implications for aiding prevention and intervention efforts. Specifically, drinking motivations are not static and can be modified through treatment. One recent study displayed that combined cognitive behavioral therapy (CBT) and behavioral motivational interviewing successfully reduced drinking-to-cope motives among depressed youth (Curtiss et al., 2021). Additionally, among patients undergoing treatment for AUD, those with enhanced drinking-to-cope motivations displayed a decrease in frequency of days spent drinking and binge episodes following treatment with CBT (Anker et al., 2016). These studies display that coping motivations are a valuable target for intervention to decrease risk for AUD. Importantly, individuals who have experienced SA represent a particularly high-risk subgroup for the development of alcohol problems (Langdon et al., 2017). Although several intervention efforts have been employed to address these issues, including early prevention and web-based intervention approaches, these programs have had mixed effects in reducing problem drinking behaviors (Dworkin et al., 2024; Stappenbeck et al., 2021; Bedard-Gilligan et al., 2019). Of note, one study among victims of SA found that a combined alcohol use and SA risk reduction intervention effectively decreased drinking-to-cope motivations, and this in turn decreased frequency of heavy episodic drinking (Gilmore & Bountress, 2016). Thus, the development of more personalized, trauma-informed approaches may be essential in reducing risk for alcohol problems in this vulnerable population.

The present study had many strengths, including a sample of youth with a wide range of complex trauma exposure as well as an objective, well-validated marker of AUD risk. The study also had several limitations. First, this study was not a comprehensive test of all trauma experiences but rather focused on interpersonal violence. Second, our SA and PA variables were created through grouping sexual assault with sexual abuse and physical abuse with interpersonal physical violence, yet we acknowledge that these trauma categories may have meaningful distinctions. Future studies with larger samples should aim to disentangle these trauma categories even further to better understand whether specific forms of trauma type may confer unique risk. We also did not investigate the impact of age at first trauma exposure. This is notable, given that there is evidence that timing of trauma exposure can significantly influence the severity of trauma-related symptoms and outcomes (A. D. Marshall, 2016; McCutcheon et al., 2010). An additional limitation is the number of statistical tests conducted in this study. Accordingly, the observed effects, particularly the interaction between sexual assault and drinking-to-cope motives, should be viewed as preliminary and replicated in future studies. The predominance of females in our sample limits the generalizability of our findings to males and precludes the examination of potential sex differences. Replication in more sex-balanced samples will also be important for additional confirmation of these findings. Future studies should continue to build off this current work to identify additional risk and protective factors that may influence the relationship between SA, coping motivations, and risk for AUD. Another limitation is that our study assessed responses to alcohol- and food-cues as opposed to consumption behaviors, which may not fully capture real-world drinking or eating reward patterns. Future studies should continue to build off this work by examining these relationships using actual behavioral measures rather than just cues. Lastly, although we used a single, parsimonious model, we did not correct for multiple predictors and interactions. Our results did not survive strict corrections for multiple comparisons and are thus preliminary.

Our results revealed that those with a history of SA and heightened drinking-to-cope motivations may be at greater risk for the development of AUD and problematic alcohol use. These findings shed light on key demographic and psychological risk factors that can contribute to heightened vulnerability for alcohol problems. As such, future research should explore if and how targeted interventions for drinking-to-cope motivations aid in preventing the onset and escalation of alcohol misuse among SA victims.

## 5. Conclusions

In sum, the present study highlights the critical role of drinking-to-cope motivations in moderating the association between SA exposure and neural reactivity to alcohol cues. Individuals with a history of SA who endorse heightened drinking-to-cope motivations display enhanced alcohol cue reactivity, reflecting potential increased risk for AUD. These results are consistent with self-medication and drinking-to-cope theories of alcohol use, which suggest that alcohol consumption can be reinforced through its capacity to alleviate distress, thereby strengthening maladaptive coping patterns and escalating risk for alcohol problems (Khantzian, 1997; Baker et al., 2004). Importantly, coping motivations have been shown to be modifiable through targeted intervention. These results underscore the value of trauma-informed prevention and intervention efforts aimed at mitigating AUD risk among SA victims. Future research should continue to explore and clarify the mechanisms that link trauma exposure, drinking-to-cope motivations, and neural alcohol cue reactivity.

## Figures and Tables

**Table 1:** Participant demographics and characteristics.

Demographics	Percentage	Demographics (Cont.)	Percentage
Age (years)	18.13	Race	
Sex (% female)	70.90%	White	63.90%
Ethnicity (% Hispanic)	10.10%	Black	12.70%
		Asian	8.20%
		American Indian or Alaskan Native	0.00%
		Biracial, Other, or Unknown	15.20%
Lifetime SCID/KSAD Diagnoses	Percentage	Current SCID/KSAD Diagnoses	Percentage
Major depressive disorder	55.70%	Major depressive disorder	8.90%
Generalized anxiety disorder	9.50%	Generalized anxiety disorder	7.60%
Social anxiety disorder	20.30%	Social anxiety disorder	13.30%
Panic disorder	0.60%	Panic disorder	0.00%
Specific phobia	2.50%	Specific phobia	1.30%
Post-traumatic stress disorder	24.10%	Post-traumatic stress disorder	4.40%
Alcohol use disorder	0.00%	Alcohol use disorder	0.00%
Substance use disorder	0.00%	Substance use disorder	0.00%

**Table 2:** Results of Hierarchical Linear Regression Analyses.

Predictors	Unstandardized β	95% CI	*t*	*p*	R^2^*_semi_*	Adjusted R^2^	ΔR^2^	ΔF	Sig ΔF
**Model 1: Trauma type and Coping Motives on Alcohol cue-elicited LPP**									
Block 1						0.037	0.055	3.004	0.032
Sex	0.218	[−0.955, 1.390]	0.367	0.714	0.03				
Age	−0.214	[−0.834, 0.405]	−0.683	0.496	−0.055				
Total PCL Score	0.054	[0.016, 0.091]	2.81	0.006 *	0.221				
Block 2						0.018	0.007	0.275	0.894
Coping Motives to Drink	0.093	[−0.358, 0.545]	0.409	0.683	0.033				
SA	0.188	[−0.997, 1.374]	0.314	0.754	0.026				
PA	−0.374	[−1.565, 0.817]	−0.621	0.536	−0.051				
DV	−0.327	[−1.670, 1.024]	−0.478	0.633	−0.04				
Block 3						0.05	0.049	2.684	0.49
SA x Coping Motives	1.113	[0.206, 2.202]	2.425	0.017 *	0.196				
DV x Coping Motives	1.311	[−0.15, 2.773]	1.773	0.078	0.145				
PA x Coping Motives	−0.123	[−1.021, 0.774]	−0.272	0.786	−0.022				
**Model 2: Trauma type and Coping Motives on Food cue-elicited LPP**									
Block 1						−0.003	0.016	0.855	0.466
Sex	−0.333	[−1.521, 0.788]	−0.572	0.532	−0.5				
Age	−0.054	[−0.665, 0.555]	−0.172	0.858	−0.014				
Total PCL Score	0.028	[−0.008, 0.067]	1.485	0.119	0.125				
Block 2			.			−0.017	0.012	0.465	0.761
Coping Motives to Drink	−0.062	[−0.506, 0.381]	−0.278	0.781	−0.023				
SA	0.466	[−0.698, 1.63]	0.792	0.43	0.065				
PA	−0.183	[−1.353, 0.986]	−0.309	0.757	−0.025				
DV	0.68	[−0.647, 2.008]	1.013	0.313	0.082				
Block 3						−0.026	0.011	0.566	0.638
SA x Coping Motives	0.453	[−0.456, 1.363]	0.985	0.326	0.081				
DV x Coping Motives	0.326	[−1.139, 1.792]	0.44	0.661	0.036				
PA x Coping Motives	−0.479	[−1.379, 0.422]	−1.05	0.295	−0.086				

Note. * = *p* < 0.05; SA = Sexual assault, PA = Physical assault, DV = Domestic violence, PCL = Posttraumatic Stress Disorder Checklist.

## Data Availability

The data supporting the findings of the present study are available from the corresponding author upon request.

## References

[B1-behavsci-16-00218] Aiken L. S., West S. G., Reno R. R. (1991). Multiple regression: Testing and interpreting interactions.

[B2-behavsci-16-00218] Almeida-Antunes N., Crego A., Carbia C., Sousa S. S., Rodrigues R., Sampaio A., Lopez-Caneda E. (2021). Electroencephalographic signatures of the binge drinking pattern during adolescence and young adulthood: A PRISMA-driven systematic review. NeuroImage: Clinical.

[B3-behavsci-16-00218] Anker J. J., Kushner M. G., Thuras P., Menk J., Unruh A. S. (2016). Drinking to cope with negative emotions moderates alcohol use disorder treatment response in patients with co-occurring anxiety disorder. Drug and Alcohol Dependence.

[B4-behavsci-16-00218] Baker T. B., Piper M. E., McCarthy D. E., Majeskie M. R., Fiore M. C. (2004). Addiction motivation reformulated: An affective processing model of negative reinforcement. Psychological Review.

[B5-behavsci-16-00218] Bartholow B. D., Henry E. A., Lust S. A. (2007). Effects of alcohol sensitivity on P3 event-related potential reactivity to alcohol cues. Psychology of Addictive Behaviors.

[B6-behavsci-16-00218] Basile K. C., Smith S. G., Kresnow M., Khatiwada S., Leemis R. W. (2022). The national intimate partner and sexual violence survey: 2016/2017 report on sexual violence.

[B7-behavsci-16-00218] Bedard-Gilligan M., Masters N. T., Ojalehto H., Simpson T. L., Stappenbeck C., Kaysen D. (2019). Refinement and pilot testing of a brief, early intervention for PTSD and alcohol use following sexual assault. Cognitive and Behavioral Practice.

[B8-behavsci-16-00218] Berenz E. C., Cho S. B., Overstreet C., Kendler K., Amstadter A. B., Dick D. M. (2016). Longitudinal investigation of interpersonal trauma exposure and alcohol use trajectories. Addictive Behaviors.

[B9-behavsci-16-00218] Berzofsky M., Krebs C., Langton L., Planty M., Smiley-McDonald H. (2013). Female victims of sexual violence, 1994–2010.

[B10-behavsci-16-00218] Bitsoih J., Patock-Peckham J. A., Canning J. R., Ong A., Becerra A., Broussard M. (2023). Do coping motives and perceived impaired control mediate the indirect links from childhood trauma facets to alcohol-related problems?. Behavioral Sciences.

[B11-behavsci-16-00218] Bountress K. E., Cusack S. E., Sheerin C. M., Hawn S., Dick D. M., Kendler K. S., Amstadter A. B. (2019). Alcohol consumption, interpersonal trauma, and drinking to cope with trauma-related distress: An auto-regressive, cross-lagged model. Psychology of Addictive Behaviors.

[B12-behavsci-16-00218] Bravo A. J., Pearson M. R. (2017). In the process of drinking to cope among college students: An examination of specific vs. global coping motives for depression and anxiety symptoms. Addictive Behaviors.

[B13-behavsci-16-00218] Brown S. B. R. E., van Steenbergen H., Band G. P. H., de Rover M., Nieuwenhuis S. (2012). Functional significance of the emotion-related late positive potential. Frontiers in Human Neuroscience.

[B14-behavsci-16-00218] Bryant R. A., Wade D., Creamer M., Silove D., McFarlane A., Forbes D., O’Donnell M., Rees S., Lockwood E., Phelps A., Chapman C., Slade T., Mills K., Teesson M. (2013). Trauma at the hands of another: Distinguishing PTSD patterns following intimate and nonintimate interpersonal and noninterpersonal trauma in a nationally representative sample. The Journal of Clinical Psychiatry.

[B15-behavsci-16-00218] Cal Poly Humboldt (2025). Supporting survivors: Educational resources—Statistics.

[B16-behavsci-16-00218] Carliner H., Keyes K. M., McLaughlin K. A., Meyers J. L., Dunn E. C., Martins S. S. (2016). Childhood trauma and illicit drug use in adolescence: A population-based national comorbidity survey replication–adolescent supplement study. Journal of the American Academy of Child & Adolescent Psychiatry.

[B17-behavsci-16-00218] Carter B. L., Tiffany S. T. (1999). Meta-analysis of cue-reactivity in addiction research. Addiction.

[B18-behavsci-16-00218] Chambers W. J., Puig-Antich J., Hirsch M., Paez P., Ambrosini P. J., Tabrizi M. A., Davies M. (1985). The assessment of affective disorders in children and adolescents by semistructured interview: Test-retest reliability of the schedule for affective disorders and schizophrenia for school-age children, present episode version. Archives of General Psychiatry.

[B19-behavsci-16-00218] Chen C. M., Yoon Y.-H. (2021). Surveillance report #116: Trends in underage drinking in the United States, 1999–2019.

[B20-behavsci-16-00218] Chung T., Creswell K. G., Bachrach R., Clark D. B., Martin C. S. (2018). Adolescent binge drinking: Developmental context and opportunities for prevention. Alcohol Research: Current Reviews.

[B21-behavsci-16-00218] Cole J., Sprang G., Lee R., Cohen J. (2016). The trauma of commercial sexual exploitation of youth: A comparison of CSE victims to sexual abuse victims in a clinical sample. Journal of Interpersonal Violence.

[B22-behavsci-16-00218] Conger J. J. (1956). II. Reinforcement theory and the dynamics of alcoholism. Quarterly Journal of Studies on Alcohol.

[B23-behavsci-16-00218] Cooper M. L. (1994). Motivations for alcohol use among adolescents: Development and validation of a four-factor model. Psychological Assessment.

[B24-behavsci-16-00218] Cooper M. L., Frone M. R., Russell M., Mudar P. (1995). Drinking to regulate positive and negative emotions: A motivational model of alcohol use. Journal of Personality and Social Psychology.

[B25-behavsci-16-00218] Cooper M. L., Kuntsche E., Levitt A., Barber L. L., Wolf S., Sher K. J. (2016). Motivational models of substance use: A review of theory and research on motives for using alcohol, marijuana, and tobacco. The Oxford handbook of substance use and substance use disorders.

[B26-behavsci-16-00218] Corbin W. R., Farmer N. M., Nolen-Hoekesma S. (2013). Relations among stress, coping strategies, coping motives, alcohol consumption and related problems: A mediated moderation model. Addictive Behaviors.

[B27-behavsci-16-00218] Covey H. C., Grubb L. M., Franzese R. J., Menard S. (2020). Adolescent exposure to violence and adult anxiety, depression, and PTSD. Criminal Justice Review.

[B28-behavsci-16-00218] Cox W. M., Klinger E. (1988). A motivational model of alcohol use. Journal of Abnormal Psychology.

[B29-behavsci-16-00218] Curtiss J. E., Wallace B., Fisher L. B., Nyer M., Jain F., Cusin C., Pedrelli P. (2021). Change processes in cognitive behavioral therapy and motivational interviewing for depression and heavy alcohol use: A network approach. Journal of Affective Disorders Reports.

[B30-behavsci-16-00218] Delorme A., Makeig S. (2004). EEGLAB: An open source toolbox for analysis of single-trial EEG dynamics including independent component analysis. Journal of Neuroscience Methods.

[B31-behavsci-16-00218] Demos K. E., Kelley W. M., Heatherton T. F. (2011). Dietary restraint violations influence reward responses in nucleus accumbens and amygdala. Journal of Cognitive Neuroscience.

[B32-behavsci-16-00218] Dworkin E. R. (2020). Risk for mental disorders associated with sexual assault: A meta-analysis. Trauma, Violence, & Abuse.

[B33-behavsci-16-00218] Dworkin E. R., Menon S. V., Bystrynski J., Allen N. E. (2017). Sexual assault victimization and psychopathology: A review and meta-analysis. Clinical Psychology Review.

[B34-behavsci-16-00218] Dworkin E. R., Schallert M., Lee C. M., Kaysen D. (2024). Pilot randomized clinical trial of an app-based early intervention to reduce PTSD and alcohol use following sexual assault. Psychological Trauma: Theory, Research, Practice, and Policy.

[B35-behavsci-16-00218] Finkelhor D., Turner H. A., Shattuck A., Hamby S. L. (2015). Prevalence of childhood exposure to violence, crime, and abuse. JAMA Pediatrics.

[B36-behavsci-16-00218] First M. B., Williams J. B. W., Karg R. S., Spitzer R. L. (2015). Structured clinical interview for DSM-5-research version (SCID-5 for DSM-5, research version; SCID-5-RV).

[B37-behavsci-16-00218] Foster D. W., Young C. M., Steers M. L. N., Quist M. C., Bryan J. L., Neighbors C. (2014). Tears in your beer: Gender differences in coping drinking motives, depressive symptoms and drinking. International Journal of Mental Health and Addiction.

[B38-behavsci-16-00218] Geda D. W., Stangl B. L., Arsenault A., Thompson M. F., Schwandt M. L., Goldman D., Ramchandani V. A., Diazgranados N., Luk J. W. (2024). Drinking motives link positive and negative life events to problematic alcohol use during the COVID-19 pandemic: A longitudinal study. Alcohol and Alcoholism.

[B39-behavsci-16-00218] Gilmore A. K., Bountress K. E. (2016). Reducing drinking to cope among heavy episodic drinking college women: Secondary outcomes of a web-based combined alcohol use and sexual assault risk reduction intervention. Addictive Behaviors.

[B40-behavsci-16-00218] Grant V. V., Stewart S. H., O’Connor R. M., Blackwell E., Conrod P. J. (2007). Psychometric evaluation of the five-factor modified drinking motives questionnaire—Revised in undergraduates. Addictive Behaviors.

[B41-behavsci-16-00218] Haering S., Meyer C., Schulze L., Conrad E., Blecker M. K., El-Haj-Mohamad R., Geiling A., Klusmann H., Schumacher S., Knaevelsrud C., Engel S. (2024). Sex and gender differences in risk factors for posttraumatic stress disorder: A systematic review and meta-analysis of prospective studies. Journal of Psychopathology and Clinical Science.

[B42-behavsci-16-00218] Hajcak G., Dunning J. P., Foti D. (2009). Motivated and controlled attention to emotion: Time-course of the late positive potential. Clinical Neurophysiology.

[B43-behavsci-16-00218] Harrison P. A., Fulkerson J. A., Beebe T. J. (1997). Multiple substance use among adolescent physical and sexual abuse victims. Child Abuse & Neglect.

[B44-behavsci-16-00218] Hawn S. E., Bountress K. E., Sheerin C. M., Dick D. M., Amstadter A. B. (2020a). Trauma-related drinking to cope: A novel approach to the self-medication model. Psychology of Addictive Behaviors.

[B45-behavsci-16-00218] Hawn S. E., Cusack S. E., Amstadter A. B. (2020b). A systematic review of the self-medication hypothesis in the context of posttraumatic stress disorder and comorbid problematic alcohol use. Journal of Traumatic Stress.

[B46-behavsci-16-00218] Herrmann M., Weijers H.-G., Wiesbeck G. A., Aranda D., Böning J., Fallgatter A. J. (2000). Event-related potentials and cue-reactivity in alcoholism. Alcoholism: Clinical and Experimental Research.

[B47-behavsci-16-00218] Jenkins K. C., Toleson S., House A., Kreutzer K., Phan K. L., Gorka S. M. (2025). Neural alcohol cue reactivity as a risk factor for future drinking in youth with limited alcohol exposure. Alcohol: Clinical and Experimental Research.

[B48-behavsci-16-00218] Kaag A. M., Wiers R. W., Vries T. J., Pattij T., Goudriaan A. E. (2019). Striatal alcohol cue-reactivity is stronger in male than female problem drinkers. European Journal of Neuroscience.

[B49-behavsci-16-00218] Kang W. (2023). Understanding the associations between personality traits and the frequency of alcohol intoxication in young males and females: Findings from the United Kingdom. Acta Psychologica.

[B50-behavsci-16-00218] Khantzian E. J. (1997). The self-medication hypothesis of substance use disorders: A reconsideration and recent applications. Harvard Review of Psychiatry.

[B51-behavsci-16-00218] Kohen C. B., Cofresí R. U., Piasecki T. M., Bartholow B. D. (2024). Predictive utility of the P3 event-related potential (ERP) response to alcohol cues for ecologically assessed alcohol craving and use. Addiction Biology.

[B52-behavsci-16-00218] Koob G., Le Moal M. (2001). Drug addiction, dysregulation of reward, and allostasis. Neuropsychopharmacology.

[B53-behavsci-16-00218] Kothe C. A., Makeig S. (2013). BCILAB: A platform for brain–computer interface development. Journal of Neural Engineering.

[B54-behavsci-16-00218] Lang P. J. (2005). International affective picture system (IAPS): Affectiveratings of pictures and instruction manual *(Technical Report A-6)*.

[B55-behavsci-16-00218] Langdon K. J., Rubin A., Brief D. J., Enggasser J. L., Roy M., Solhan M., Helmuth E., Rosenbloom D., Keane T. M. (2017). Sexual traumatic event exposure, posttraumatic stress symptomatology, and alcohol misuse among women: A critical review of the empirical literature. Clinical Psychology: Science and Practice.

[B56-behavsci-16-00218] Levin Y., Bar-Or R. L., Forer R., Vaserman M., Kor A., Lev-Ran S. (2021). The association between type of trauma, level of exposure and addiction. Addictive Behaviors.

[B57-behavsci-16-00218] Littel M., Euser A. S., Munafò M. R., Franken I. H. A. (2012). Electrophysiological indices of biased cognitive processing of substance-related cues: A meta-analysis. Neuroscience & Biobehavioral Reviews.

[B58-behavsci-16-00218] Lopez-Calderon J., Luck S. J. (2014). ERPLAB: An open-source toolbox for the analysis of event-related potentials. Frontiers in Human Neuroscience.

[B59-behavsci-16-00218] Marshall A. D. (2016). Developmental timing of trauma exposure relative to puberty and the nature of psychopathology among adolescent girls. Journal of the American Academy of Child & Adolescent Psychiatry.

[B60-behavsci-16-00218] Marshall E. J. (2014). Adolescent alcohol use: Risks and consequences. Alcohol and Alcoholism.

[B61-behavsci-16-00218] McCutcheon V. V., Sartor C. E., Pommer N. E., Bucholz K. K., Nelson E. C., Madden P. A. F., Heath A. C. (2010). Age at trauma exposure and PTSD risk in young adult women. Journal of Traumatic Stress.

[B62-behavsci-16-00218] McLaughlin K. A., Lambert H. K. (2017). Child trauma exposure and psychopathology: Mechanisms of risk and resilience. Current Opinion in Psychology.

[B63-behavsci-16-00218] Messman-Moore T., Ward R. M., Zerubavel N., Chandley R. B., Barton S. N. (2014). Emotion dysregulation and drinking to cope as predictors and consequences of alcohol-involved sexual assault. Journal of Interpersonal Violence.

[B64-behavsci-16-00218] Moran T. P., Jendrusina A. A., Moser J. S. (2013). The psychometric properties of the late positive potential during emotion processing and regulation. Brain Research.

[B65-behavsci-16-00218] Muehlenhard C. L., Peterson Z. D., Humphreys T. P., Jozkowski K. N. (2017). Evaluating the one-in-five statistic: Women’s risk of sexual assault while in college. The Journal of Sex Research.

[B66-behavsci-16-00218] Noudali S. N., Patock-Peckham J. A., Berberian S. L., Belton D. A., Campbell L. E., Infurna F. J. (2022). Does insomnia mediate the link between childhood trauma and impaired control over drinking, alcohol use, and related problems?. Addictive Behaviors Reports.

[B67-behavsci-16-00218] Park C. L., Levenson M. R. (2002). Drinking to cope among college students: Prevalence, problems and coping processes. Journal of Studies on Alcohol.

[B68-behavsci-16-00218] Patock-Peckham J. A., Belton D. A., D’Ardenne K., Tein J. Y., Bauman D. C., Infurna F. J., Sanabria F., Curtis J., Morgan-Lopez A. A., McClure S. M. (2020). Dimensions of childhood trauma and their direct and indirect links to PTSD, impaired control over drinking, and alcohol-related-problems. Addictive Behaviors Reports.

[B69-behavsci-16-00218] Pynoos R. S., Steinberg A. M., Layne C. M., Liang L. J., Vivrette R. L., Briggs E. C., Kisiel C., Habib M., Belin T. R., Fairbank J. A. (2014). Modeling constellations of trauma exposure in the national child traumatic stress network core data set. Psychological Trauma: Theory, Research, Practice, and Policy.

[B70-behavsci-16-00218] Radoman M., McGowan C., Heilner E., Lacadie C., Sinha R. (2024). Neural responses to stress and alcohol cues in individuals with pain with and without alcohol use disorder. Addiction Biology.

[B71-behavsci-16-00218] Rich S. L., Wilson J. K., Robertson A. A. (2016). The impact of abuse trauma on alcohol and drug use: A study of high-risk incarcerated girls. Journal of Child & Adolescent Substance Abuse.

[B72-behavsci-16-00218] Sartor C. E., Agrawal A., McCutcheon V. V., Duncan A. E., Lynskey M. T. (2008). Disentangling the complex association between childhood sexual abuse and alcohol-related problems: A review of methodological issues and approaches. Journal of Studies on Alcohol and Drugs.

[B73-behavsci-16-00218] Schneekloth T. D., Biernacka J. M., Hall-Flavin D. K., Karpyak V. M., Frye M. A., Loukianova L. L., Stevens S. R., Drews M. S., Geske J. R., Mrazek D. A. (2012). Alcohol craving as a predictor of relapse. The American Journal on Addictions.

[B74-behavsci-16-00218] Shuai R., Anker J. J., Bravo A. J., Kushner M. G., Hogarth L. (2022). Risk pathways contributing to the alcohol harm paradox: Socioeconomic deprivation confers susceptibility to alcohol dependence via greater exposure to aversive experience, internalizing symptoms and drinking to cope. Frontiers in Behavioral Neuroscience.

[B75-behavsci-16-00218] Simpson T. L., Stappenbeck C. A., Luterek J. A., Lehavot K., Kaysen D. L. (2014). Drinking motives moderate daily relationships between PTSD symptoms and alcohol use. Journal of Abnormal Psychology.

[B76-behavsci-16-00218] Sinha R. (2011). Effects of adrenal sensitivity, stress- and cue-induced craving, and anxiety on subsequent alcohol relapse and treatment outcomes. Archives of General Psychiatry.

[B77-behavsci-16-00218] Smit K., Voogt C., Otten R., Kleinjan M., Kuntsche E. (2022). Why adolescents engage in early alcohol use: A study of drinking motives. Experimental and Clinical Psychopharmacology.

[B78-behavsci-16-00218] Smith H. L., Summers B. J., Dillon K. H., Cougle J. R. (2016). Is worst-event trauma type related to PTSD symptom presentation and associated features?. Journal of Anxiety Disorders.

[B79-behavsci-16-00218] Smith K. Z., Smith P. H., Grekin E. R. (2014). Childhood sexual abuse, distress, and alcohol-related problems: Moderation by drinking to cope. Psychology of Addictive Behaviors.

[B80-behavsci-16-00218] Stangeland H., Aakvaag H. F., Baumann-Larsen M., Wentzel-Larsen T., Storheim K., Zwart J. A., Dyb G., Stensland S. Ø. (2023). Problematic alcohol use in young adults exposed to childhood trauma: The trøndelag health (HUNT) study. Journal of Traumatic Stress.

[B81-behavsci-16-00218] Stapinski L. A., Edwards A. C., Hickman M., Araya R., Teesson M., Newton N. C., Kendler K. S., Heron J. (2016). Drinking to cope: A latent class analysis of coping motives for alcohol use in a large cohort of adolescents. Prevention Science.

[B82-behavsci-16-00218] Stappenbeck C. A., Gulati N. K., Jaffe A. E., Blayney J. A., Kaysen D. (2021). Initial efficacy of a web-based alcohol and emotion regulation intervention for college women with sexual assault histories. Psychology of Addictive Behaviors.

[B83-behavsci-16-00218] Stappenbeck C. A., Hassija C. M., Zimmerman L., Kaysen D. (2015). Sexual assault related distress and drinking: The influence of daily reports of social support and coping control. Addictive Behaviors.

[B84-behavsci-16-00218] Steinberg A. M., Brymer M. J., Decker K. B., Pynoos R. S. (2004). the university of california at los angeles post-traumatic stress disorder reaction index. Current Psychiatry Reports.

[B85-behavsci-16-00218] Ullman S. E. (2003). A critical review of field studies on the link of alcohol and adult sexual assault in women. Aggression and Violent Behavior.

[B86-behavsci-16-00218] Vafaie N., Kober H. (2022). Association of drug cues and craving with drug use and relapse: A systematic review and meta-analysis. JAMA Psychiatry.

[B87-behavsci-16-00218] Wagner D. D., Boswell R. G., Kelley W. M., Heatherton T. F. (2012). Inducing negative affect increases the reward value of appetizing foods in dieters. Journal of Cognitive Neuroscience.

[B88-behavsci-16-00218] Weathers F. W., Litz B. T., Keane T. M., Palmieri P. A., Marx B. P., Schnurr P. P. (2013). The PTSD checklist for DSM-5 (PCL-5)—LEC-5 and extended criterion A *[Measurement instrument]*.

[B89-behavsci-16-00218] Windle M., Windle R. C. (2015). A prospective study of stressful events, coping motives for drinking, and alcohol use among middle-aged adults. Journal of Studies on Alcohol and Drugs.

[B90-behavsci-16-00218] Zaso M. J., Read J. P., Colder C. R. (2023). Coping-motivated escalations in adolescent alcohol problems following early adversity. Psychology of Addictive Behaviors.

